# Comparison of relapse of orthodontic treatment following aligner versus conventional fixed appliance treatment. A systematic review

**DOI:** 10.4317/jced.61520

**Published:** 2024-05-01

**Authors:** Christian-Fabrizio Regalado-Bazán, Andrea-Carolina Espichan-Salazar, Luis-Ernesto Arriola-Guillén

**Affiliations:** 1 School of Dentistry, Universidad Científica del Sur, Lima, Perú; 2 Ph.D. and Associate Professor of the Division of Orthodontics and Division of Oral and Maxillofacial Radiology, School of Dentistry, Universidad Científica del Sur, Lima, Perú

## Abstract

**Background:**

Aligners are an alternative that are currently being widely used in orthodontic treatment, however, post-treatment relapse following aligner versus conventional treatment has not been compared. The objective of this study was to compare post-treatment relapse of orthodontic treatment with dental aligners versus conventional fixed orthodontics through a systematic review.

**Material and Methods:**

An exhaustive search was carried out in the MEDLINE (via PubMed), EBSCO, SCOPUS and EMBASE databases up to September 30, 2023. A total of 522 articles were found and after applying the selection criteria, the full texts of 24 articles were chosen for evaluation. At the end of the evaluation, only 3 studies were considered, two observational studies and one randomized clinical trial. The Newcastle Ottawa and Risk of bias (ROB-2) tools were used to assess the risk of bias.

**Results:**

Of the three studies, only one (evaluated three years post-orthodontic treatment) identified significant differences in the frequency of relapse following total, maxillary and mandibular anterior alignment using conventional orthodontics. The other two studies (one evaluated at six months and the other two years post-orthodontic treatment) did not show statistically significant differences (*p*>0.05). In addition, the two latter studies reported slight relapse related to detachment of the fixed retainer.

**Conclusions:**

The current evidence available indicates a lack of well-designed studies comparing post-treatment orthodontic relapse following the use of dental aligners versus conventional fixed orthodontic treatment, and the studies published to date have a high or moderate risk of bias. However, there are apparently no significant differences in frequency of relapse between the two types of treatment, suggesting similar stability results. Nonetheless, more well-designed studies are required to confirm this observation.

** Key words:**Aligners, brackets, relapse, orthodontic.

## Introduction

Nowadays, maintaining teeth in the correct position after orthodontic treatment remains a challenge for orthodontists and is a great desire for patients (1). Teeth are prone to returning to their initial position due to occlusal, gingival and/or periodontal factors, which are influenced by growth and craniofacial development (2,3). Orthodontic relapse is defined as the return of the treated teeth to the initial position, with partial or total recurrence (4). Thus, orthodontists must be certain about the etiology of post-treatment relapse and be familiar with and know the pros and cons of the various types of containment in order to reduce the probability of relapse (5-7). Moreover, it is also important for patients to understand that teeth may undergo changes post-orthodontic treatment due to factors that seek to return the teeth to their initial position (8).

Treatment with orthodontic aligners has significantly increased over time, which, in turn, has aroused the interest of a large part of the population because it is a more esthetic treatment alternative that is more accepted by patients, facilitates oral hygiene and allows for efficient alignment compared to conventional orthodontics (9,10). In short, aligners offer a more esthetic alternative, and the results of this technique have improved in different malocclusions (11). Tooth movement with this method is incremental through the use of various aligners, which progressively reposition the teeth. These movements are caused by two mechanisms that include the molding effect consisting of moving the teeth to the shape of the aligner used, and the use of auxiliary elements, which are used to provide greater predictability in tooth movements (12-14).

In general, the use of a containment appliance at the end of treatment is indispensable, and the decision as to which appliance to use, whether removable or fixed, should be mutually agreed upon between the orthodontist and the patient in order to achieve greater efficiency. However, there is currently a high degree of uncertainty regarding the development of relapse that may occur after orthodontic treatment with dental aligners (15).

On the other hand, most of the cases related to the use of aligners described in the literature report difficulties in correcting extrusion, rotation or protrusion as well as the need for refinement in the middle of treatment. In addition, aligners are often used in conjunction with fixed appliances, and sometimes retreatment with brackets is necessary to achieve better results (16). It has also been described that the efficacy of aligners increases in mild to moderate malocclusions, presenting less efficient results when applied in cases with severe malocclusions (17,18). Another study has described orthodontic treatments that combine the use of aligners and computer-guided piezocision, evaluating the follow-up over time and reporting relapse that ranges from 0.2 mm to 0.25 mm (18).

For this reason, it is essential for orthodontists to be aware of the prevalence of relapse with the use of the popular method of aligners compared to conventional orthodontics taking into account that meshing with aligners may not be as ideal as in fixed orthodontic treatment, and consequently, there is increased risk of relapse (19). Therefore, the aim of this study was to compare relapse after orthodontic treatment with dental aligners versus conventional orthodontics with brackets, through a systematic review.

## Material and Methods

-Protocol and registration:

The present systematic review was registered with the Research and Ethics Commission of the Universidad Científica del Sur with registration code No. PRE-8-2022-00603, and was also registered in the INPLASY database with registration code 202360097. Details of the protocol can be found at the following address: https://inplasy.com/ 

The structure of this review followed all preferred reporting element guidelines for systematic reviews and meta-analyses (PRISMA).

-Inclusion criteria 

A question was formulated with the PICOs strategy to assist in the selection of eligible studies. The eligibility criteria were as follows:

• Population: individuals with permanent dentition, older than 14 years of age, of any sex and race, with different malocclusions, who received orthodontic treatment with aligners or with brackets and were discharged using any containment appliance for 6 to 18 months.

• Intervention: Orthodontic treatment with aligners.

• Comparison: Orthodontic treatment with conventional fixed appliances with brackets.

• Outcomes: Dental relapse after orthodontic treatment as the primary outcome in total alignment, marginal ridges, buccolingual inclination, occlusal contacts, interproximal contacts, overjet, occlusal relationship, and root angulations. The percentage of relapse was also evaluated.

• Study design: Analytical studies, clinical trials, or retrospective studies, including patients with orthodontic treatment with aligners versus orthodontic treatment with brackets as comparison groups.

-Search strategies

The search strategies were applied in the following electronic databases until September 30, 2023: MEDLINE (via PubMed), EBSCO, SCOPUS and EMBASE. The search strategies were limited to humans, without restriction of publication time, in Spanish, English or Portuguese. The search terms (strategies) were developed for MEDLINE (via PubMed), SCOPUS, EBSCO and EMBASE as shown in  Table 1.

-Study selection 

**Figure 1:**
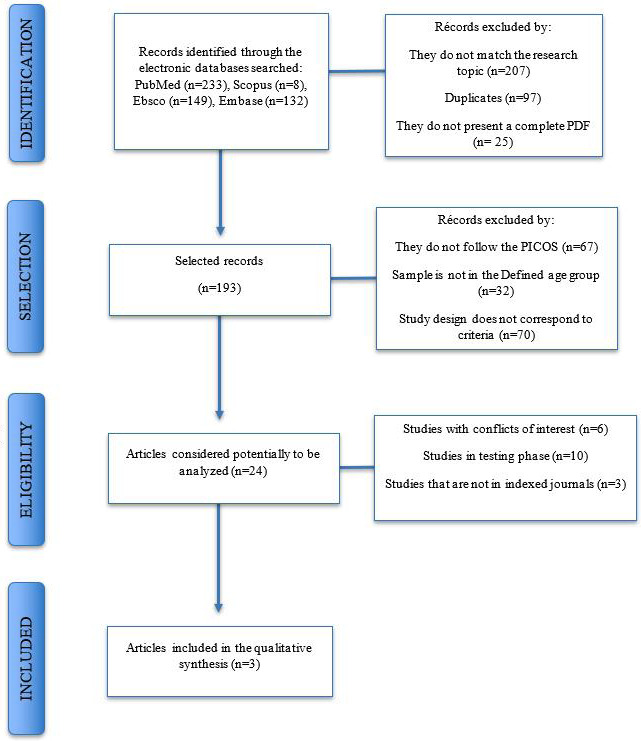
Flowchart of selected studies.

The search strategy found a total of 522 articles in the databases. After applying the inclusion criteria and elimination of duplicate articles, 24 articles were selected for evaluation of the full text. At the end of the evaluation of the articles, only 3 studies that met the selection criteria (2 prospective and 1 clinical trial) were included in this systematic review. A search flow diagram is provided in Figure 1.

-Quality assessment 

The risk of bias for observational studies was assessed using the Newcastle Ottawa scale (20). The following items were assessed: Selection, Comparability, and Outcome. The bias in each item and the overall risk of bias of the studies were considered as high or low risk information. https://www.ohri.ca/programs/clinical_epidemiology/oxford.asp 

The risk of bias for randomized clinical trials was assessed using the ROB 2 scale (21). The following items were assessed: bias due to deviations from intended interventions, bias due to loss of outcome data, bias due to measurement of outcomes, bias due to selection of reported outcomes. The bias in each item and the overall risk of bias of randomized clinical trials were considered as high, moderate, and low risk information. https://www.riskofbias.info/welcome/rob-2-0-too 

## Results

-General characteristics of the articles analyzed

 Table 2 describes the general characteristics of the studies analyzed. Of the 3 studies selected, 2 were analytical observational studies (retrospective cohort studies) (23,24) and 1 was a randomized clinical trial (25). With respect to population, the study by Lanteri *et al*. (24) (n=200) was larger compared to the studies by Kuncio *et al*. (23) (n=22) and Lin *et al*. (25) (n=54). Regarding the inclusion criteria, the 3 studies coincided in that they included patients who received orthodontic treatment with aligners (n=137) and conventional treatment (n=139), with the auxiliary diagnostic tests being similar in all three, using study models and panoramic radiographs. The studies by Kuncio *et al*. (23) and Lin *et al*. (25) coincided in the parameters for the evaluation of relapse that included assessment of total alignment, changes in marginal ridges, buccolingual inclination, occlusal contacts, interproximal contacts, overjet, occlusal relationship and root angulations, while the study by Lanteri *et al*. (24) evaluated parameters such as overjet, overbite, and midline. However, the latter study only reported total alignment in both jaws in the anterior sector.

-Risk of bias 

The studies by Kuncio *et al*. (23) and Lanteri *et al*. (24) were considered to have high risk of bias, since both articles did not present great external validity due to the sample selection process and the type of sampling. Likewise, they did not provide previous information about the patients regarding their history of orthodontic treatment. In the study by Lin *et al*. (25) the two study groups started with the same amount of crowding and similar angle malocclusions and were therefore comparable. However, in the studies by Kuncio *et al*. (23) and Lanteri *et al*. (24) there was no information regarding whether the treatment groups had the same malocclusion or the same amount of crowding at the start of treatment, leading to a high bias because the groups could not be adequately compared.

**Figure 2:**
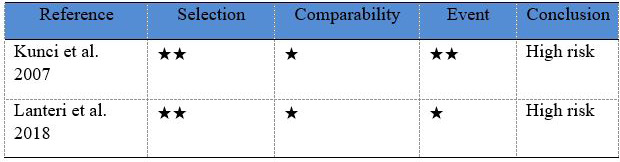
Risk of bias for the selected studies (Newcastle Ottawa).

Lanteri *et al*. (24) used digital study models to evaluate the results, while Kuncio *et al*. (23) and Lin *et al*. (25) used conventional study models, although it has been shown that there is no significant difference when comparing these two evaluation methods (26) (Fig. 2).

Regarding the follow-up period, in the study by Kuncio *et al*. (23) the follow-up period was 3 years, being 2 years in the study by Lanteri *et al*. (24) and 6 months in the study by Lin *et al*. (25). This indicates the likelihood of bias in the follow-up periods since the longer the time following the end of treatment the greater the probability of relapse.

**Figure 3:**
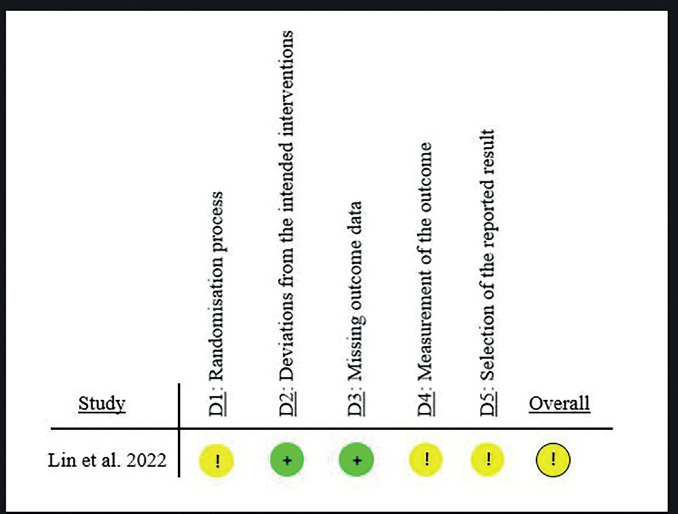
Risk of bias for the selected study (ROB 2).

The risk of bias in the study by Lin *et al*. (25) was moderate due to deficiencies in the randomization process. These authors did not specify whether patients were blinded to the randomization process. In addition, there was bias due to deviations in the interventions, since in the end all the cases with aligners required refinement with fixed appliances and thus, the comparison was really with the mixed technique. Moreover, the article did not mention the efficient use of the brackets after the end of the treatment, which could alter the interpretation of the results, (Fig. 3).

-Outcomes of the studies analyzed

 Table 3 describes the amount of relapse according to each parameter evaluated, with negative signs referring to post-treatment tooth movement (relapse). In the studies by Kuncio *et al*. (23) and Lin *et al*. (25) similar measurement parameters were evaluated, including total, anterior and posterior alignment, both at the maxillary and mandibular level, occlusal contacts and relationship, overjet, interproximal contacts and root angulations. Specifically, the results obtained by Kuncio *et al*. (23) showed a significant difference in relapse compared to the study by Lin *et al*. (25) in the following parameters: total alignment (aligners: -2.9 standard deviation [SD]:1.64); brackets: -1.36 SD:1.21), mandibular anterior alignment (aligners: -1.55 SD:1.21; brackets: -0.72 SD:0.79), maxillary anterior alignment (aligners: -1.09 SD:1.04; brackets: -0.36 SD:0.51), and maxillary anterior alignment (aligners: -1.09 SD:1.04; brackets: -0.36 SD:0.51), while in the study by Lin *et al*. (25) there were changes at 6 months after the end of treatment in the alignment parameter (aligners: 5.5±3.5; brackets: 5.75±3.0), buccolingual inclination (aligners: 2.25±1.0; brackets: 2.0±2.0); however the overall objective grading system scores in the study (OGS: aligners:12.5; brackets:14.5) showed no statistically significant differences. The study by Lanteri *et al*. (24) evaluated the percentage of relapse of the maxillary and mandibular arches both in the treatment with aligners and with brackets, resulting in relapse with both treatments during the two-year follow-up period: treatment with aligners (maxillary arch: 5% versus mandibular arch: 11%); treatment with brackets (maxillary arch: 6%, mandibular arch: 11%) (*p*>0.05). However, there was no significant difference in relapse with both aligners and brackets. Additionally, Lanteri *et al*. (24) described relapse at 1 year due to retainer loosening (< 1.5 mm) in 16 patients with orthodontic treatment with aligners and 15 undergoing orthodontic treatment with brackets, in comparison to the studies of Kuncio *et al*. (23) and Lin *et al*. (25) in which the exact number of patients presenting treatment relapse was not reported.

## Discussion

One of the most critical challenges for orthodontists is orthodontic relapse, since cases frequently begin to relapse immediately after the completion of orthodontic treatment, with this effect being more critical when there is non-compliance or deficiencies with the established containment protocol. In this sense, to avoid further relapse regardless of the orthodontic technique used (brackets or aligners), clinicians seek to finish their treatments with good intercuspation because this helps to reduce the possibility of teeth moving over time, favored by good dental meshing (21). Therefore, the purpose of this systematic review was to compare the amount and percentage of relapse after orthodontic treatment performed with dental aligners versus conventional treatment with brackets.

The post-orthodontic follow-up period was 3 years in the study by Kuncio *et al*. (23), being 2 years in that of Lanteri *et al*. (24) and a year and a half in the study by Lin *et al*. (25). In this regard, Graf *et al*. (22) described the importance of evaluation over a prolonged follow-up period, which allows more accurate evaluation of the development of relapse evidenced by changes in position produced after the removal of the orthodontic device. Nonetheless, the greatest changes are evidenced in the first months post-orthodontic treatment, with the main relapse rates occurring during the first year post-treatment.

It is important to describe the containment protocols of the three studies included in this review. In the article by Kuncio *et al*. (23) patients treated with aligners received the last aligner used as retainer in the upper and lower arches and patients treated with brackets received an essix retainer in both arches. The patients undergoing treatment with aligners in the study by Lanteri *et al*. (24) received fixed retainers in the upper and lower arch plus the last used aligner as retainer modified to fit the fixed retainer and patients treated with brackets received fixed retainers in both arches. Finally, in the study by Lin *et al*. (25) Hawley wraparound retainers were used in the upper arch and Gemini extended retainers were placed in the lower arch in patients treated with both aligners and brackets. It is clear that with the use of these retainers (an indispensable phase of orthodontic treatment) the possibility of relapse is lower than with no use at all, but it is not nil and this was evidenced by the fact that in both groups the percentage of relapse was higher with aligners (maxillary arch: 5% versus mandibular arch: 11%) versus brackets (maxillary arch: 6%, mandibular arch: 11%) in an average follow-up time of two years.

In relation to relapse post-orthodontic treatment, Kuncio *et al*. (23) reported that treatments with aligners presented greater relapse in total alignment (-2.91 aligners versus -1.36 brackets, *p*= 0.0340), which might be explained in that aligners mainly align the teeth and a good intercuspation at the end of treatment could be absent, and thus, the teeth may move more easily and in less time. However, the studies by Lanteri *et al*. (24) and Lin *et al*. (25) do not support this and described no significant difference in the amount and percentage of relapse between the two groups. The studies analyzed evaluated relapse over time by means of changes not only in the incisors but also changes considering total alignment, leveling, proximal contacts, interdental spaces, intercuspation, occlusal contacts, overjet, molar and premolar intercuspation relationship, and concluded that aligners could be considered efficient in the completion and, consequently, good intercuspation, with a lower amount and percentage of relapse, This is in agreement with Key *et al*. (19) who found that both treatments were effective in patients with some type of malocclusion: however the level of complexity at the beginning of treatment was not considered and this aspect is important since there could have been cases of less complexity with possible repercussions on the amount of relapse. Therefore, future studies comparing the complexity of the cases at the beginning of treatment should be carried out.

It should be noted that the parameters evaluated by the studies analyzed to measure relapse not only included dental alignment but also interproximal contact, root angulation, overjet, and occlusal relationship, which are good parameters to comprehensively evaluate occlusion and, consequently, relapse. In general, the disparity of the conclusions found among the three studies analyzed limits the generalization of these results and therefore no definitive position can be taken. In addition, the methodological level of the studies analyzed must be considered, with a mainly high level of risk of bias. Nevertheless, this review will likely promote the development of better methodologically designed studies.

## Conclusions

Few studies in the literature have compared relapse following post-orthodontic treatment with dental aligners versus conventional orthodontic treatments with brackets and those available have a high or moderate risk of bias. However, based on the results of the present review, there appears to be no major difference in post-orthodontic relapse between the two treatment groups, with both achieving the same results in stability. Nevertheless, this should be confirmed in further well-designed studies.

## Figures and Tables

**Table 1:** Search strategy in different databases.

Electronic database	(Key terms)
PubMed	("Relapse" OR "Recrudescence" OR "Recrudescences" OR "Recurrences" OR "Relapses") AND ("Appliance, Removable Orthodontic" OR "Appliances, Removable Orthodontic" OR "Orthodontic Appliance, Removable" OR "Removable Orthodontic Appliance" OR "Removable Orthodontic Appliances" OR "Clear Aligner Appliances" OR "Aligner Appliance, Clear" OR "Aligner Appliances, Clear" OR "Appliance, Clear Aligner" OR "Appliances, Clear Aligner" OR "Clear Aligner Appliance" OR "Clear Dental Brackets" OR "Brace, Clear Dental" OR "Brackets, Clear Dental" OR "Clear Dental Brace" OR "Dental Brace, Clear" OR "Dental Brackets, Clear" OR "Invisalign") OR Clear Aligner AND ("Fixed Orthodontic Appliances" OR "Orthodontic Appliances, Fixed" OR "Appliance, Fixed Orthodontic" OR "Appliances, Fixed Orthodontic" OR "Fixed Orthodontic Appliance" OR "Fixed Orthodontic Appliances" OR "Orthodontic Appliance, Fixed" OR "Fixed Functional Appliances" OR "Appliance, Fixed Functional" OR "Appliances, Fixed Functional" OR "Fixed Functional Appliance" OR "Functional Appliance, Fixed" OR "Functional Appliances, Fixed" OR "Fixed Retainer" OR "Fixed Retainers" OR "Retainer, Fixed" OR "Retainers, Fixed" OR "Bonded Retainer" OR "Bonded Retainers" OR "Retainer, Bonded" OR "Retainers, Bonded" OR "Fixed Appliances" OR "Appliance, Fixed" OR "Appliances, Fixed" OR "Fixed Appliance" OR "Permanent Retainer" OR "Permanent Retainers" OR "Retainer, Permanent" OR "Retainers, Permanent")
Scopus	("Relapse" OR "Recrudescence" OR "Recrudescences" OR "Recurrences" OR "Relapses") AND ("Appliance, Removable Orthodontic" OR "Appliances, Removable Orthodontic" OR "Orthodontic Appliance, Removable" OR "Removable Orthodontic Appliance" OR "Removable Orthodontic Appliances" OR "Clear Aligner Appliances" OR "Aligner Appliance, Clear" OR "Aligner Appliances, Clear" OR "Appliance, Clear Aligner" OR "Appliances, Clear Aligner" OR "Clear Aligner Appliance" OR "Clear Dental Brackets" OR "Brace, Clear Dental" OR "Brackets, Clear Dental" OR "Clear Dental Brace" OR "Dental Brace, Clear" OR "Dental Brackets, Clear" OR "Invisalign") OR Clear Aligner AND ("Fixed Orthodontic Appliances" OR "Orthodontic Appliances, Fixed" OR "Appliance, Fixed Orthodontic" OR "Appliances, Fixed Orthodontic" OR "Fixed Orthodontic Appliance" OR "Fixed Orthodontic Appliances" OR "Orthodontic Appliance, Fixed" OR "Fixed Functional Appliances" OR "Appliance, Fixed Functional" OR "Appliances, Fixed Functional" OR "Fixed Functional Appliance" OR "Functional Appliance, Fixed" OR "Functional Appliances, Fixed" OR "Fixed Retainer" OR "Fixed Retainers" OR "Retainer, Fixed" OR "Retainers, Fixed" OR "Bonded Retainer" OR "Bonded Retainers" OR "Retainer, Bonded" OR "Retainers, Bonded" OR "Fixed Appliances" OR "Appliance, Fixed" OR "Appliances, Fixed" OR "Fixed Appliance" OR "Permanent Retainer" OR "Permanent Retainers" OR "Retainer, Permanent" OR "Retainers, Permanent")
Ebsco	("Relapse" OR "Recrudescence" OR "Recrudescences" OR "Recurrences" OR "Relapses") AND ("Appliance, Removable Orthodontic" OR "Appliances, Removable Orthodontic" OR "Orthodontic Appliance, Removable" OR "Removable Orthodontic Appliance" OR "Removable Orthodontic Appliances" OR "Clear Aligner Appliances" OR "Aligner Appliance, Clear" OR "Aligner Appliances, Clear" OR "Appliance, Clear Aligner" OR "Appliances, Clear Aligner" OR "Clear Aligner Appliance" OR "Clear Dental Brackets" OR "Brace, Clear Dental" OR "Brackets, Clear Dental" OR "Clear Dental Brace" OR "Dental Brace, Clear" OR "Dental Brackets, Clear" OR "Invisalign") OR Clear Aligner AND ("Fixed Orthodontic Appliances" OR "Orthodontic Appliances, Fixed" OR "Appliance, Fixed Orthodontic" OR "Appliances, Fixed Orthodontic" OR "Fixed Orthodontic Appliance" OR "Fixed Orthodontic Appliances" OR "Orthodontic Appliance, Fixed" OR "Fixed Functional Appliances" OR "Appliance, Fixed Functional" OR "Appliances, Fixed Functional" OR "Fixed Functional Appliance" OR "Functional Appliance, Fixed" OR "Functional Appliances, Fixed" OR "Fixed Retainer" OR "Fixed Retainers" OR "Retainer, Fixed" OR "Retainers, Fixed" OR "Bonded Retainer" OR "Bonded Retainers" OR "Retainer, Bonded" OR "Retainers, Bonded" OR "Fixed Appliances" OR "Appliance, Fixed" OR "Appliances, Fixed" OR "Fixed Appliance" OR "Permanent Retainer" OR "Permanent Retainers" OR "Retainer, Permanent" OR "Retainers, Permanent")
Embase	("Relapse" OR "Recrudescence" OR "Recrudescences" OR "Recurrences" OR "Relapses") AND ("Appliance, Removable Orthodontic" OR "Appliances, Removable Orthodontic" OR "Orthodontic Appliance, Removable" OR "Removable Orthodontic Appliance" OR "Removable Orthodontic Appliances" OR "Clear Aligner Appliances" OR "Aligner Appliance, Clear" OR "Aligner Appliances, Clear" OR "Appliance, Clear Aligner" OR "Appliances, Clear Aligner" OR "Clear Aligner Appliance" OR "Clear Dental Brackets" OR "Brace, Clear Dental" OR "Brackets, Clear Dental" OR "Clear Dental Brace" OR "Dental Brace, Clear" OR "Dental Brackets, Clear" OR "Invisalign") OR Clear Aligner AND ("Fixed Orthodontic Appliances" OR "Orthodontic Appliances, Fixed" OR "Appliance, Fixed Orthodontic" OR "Appliances, Fixed Orthodontic" OR "Fixed Orthodontic Appliance" OR "Fixed Orthodontic Appliances" OR "Orthodontic Appliance, Fixed" OR "Fixed Functional Appliances" OR "Appliance, Fixed Functional" OR "Appliances, Fixed Functional" OR "Fixed Functional Appliance" OR "Functional Appliance, Fixed" OR "Functional Appliances, Fixed" OR "Fixed Retainer" OR "Fixed Retainers" OR "Retainer, Fixed" OR "Retainers, Fixed" OR "Bonded Retainer" OR "Bonded Retainers" OR "Retainer, Bonded" OR "Retainers, Bonded" OR "Fixed Appliances" OR "Appliance, Fixed" OR "Appliances, Fixed" OR "Fixed Appliance" OR "Permanent Retainer" OR "Permanent Retainers" OR "Retainer, Permanent" OR "Retainers, Permanent")

**Table 2:** General characteristics of the articles analyzed (n=3).

Author (year)	Study design	Population	Inclusion criteria	Patients' initial condition	Methods	Measurements	Treatment duration
Kuncio et al. (23), 2007	Prospective cohort study	22 patients (exposed group: 11, non-exposed (control) group: 11)	- Patients with conventional orthodontic treatment versus aligners. - Patients who have worn any type of retainer full time for at least 6 months.	Dentoalveolar discrepancies (crowding)	-Panoramic X-Ray -Study models	Changes in the following parameters: - Total alignment - Marginal ridges - Bucco lingual inclination - Occlusal contacts - Occlusal relationship - Projection - Interproximal contacts - Root angulation	3-year follow-up
Lanteri et al. (24), 2018	Retrospective cohort study	200 patients (exposed group: 100, non-exposed (control) group: 100)	- Patients whose treatment was in both arches and completed before June 2015. - Record of digital plaster models available before treatments, before refinements and after appliance removal. - Patients over 14 years of age with complete permanent dentition. - Patients who did not have any extraction or orthognathic surgery.	Dentoalveolar discrepancies	-Digital study models	Changes in the following parameters: Maxillary and mandibular anterior segments. Left and right buccal oclussion Overjet Overbite Midlines	2-year follow-up
Lin et al. (25), 2022	Randomized controlled trial	54 patients (experimental group: 26, control group: 28)	- Patients with Class I molar and canine relationships. - Patients who have not undergone extractions. -- Patients with mandibular crowding less than 4 mm.	Class I malocclusion	- Records of study models -Panoramic X-Ray	Changes in the following parameters: - Total alignment - Marginal ridges - Bucco-lingual inclination - Occlusal contacts. - Overjet - Interproximal contacts - Root angulation -Occlusal relationship	Follow-up for 1 year 7 months

**Table 3:** Outcomes of the studies analyzed.

Author (year)	Relapse in both groups		Prevalence by subgroups (no. of persons who presented recurrence)		Bivariate	Conclusion
	Aligners	Brackets	Aligners	Brackets	p-value	-
Kuncio et al. (23) 2007	Total alignment: -2.91 (SD 1.64)	-1.36 (SD 1.21)	-	-	.0340	Patients treated with Invisalign relapsed more frequently than those treated with conventional fixed appliances.
	Maxillary anterior alignment: -1.09 (SD 1.04)	-0,36 (SD 0,51)			.0859	
	Maxillary posterior alignment: -0.27 (SD 0.64)	-0.18 (SD 0.40)			.9615	
	Mandibular anterior alignment: -1.55 (SD 1.21)	-0.72 (SD 0.79)			.1133	
	Posterior mandibular alignment: -0.09 (SD 0.54)	-0.09 (SD 0.30)			1.0000	
	Marginal ridges: 0.45 (SD 2.30)	-1.00 (SD 2.00)			.4626	
	Buccolingual inclination: -0.18 (SD 1.40)	0.09 (SD 2.30)			.8644	
	Occlusal contacts: 0.36 (SD 4.63)	1.91 (SD 3.36)			.6244	
	Occlusal relationships: 0.36 (SD 4.18)	0.55 (SD 3.47)			.2083	
	Overjet: 0.91 (SD 3.78)	0.90 (SD 2.43)			.7676	
	Interproximal contacts: 0.18 (SD 0.60)	0.64 (SD 1.21)			.3670	
	Root angulation: 0.09 (SD 0.30)	-0.36 (SD 0.81)			.1169	
Lanteri et al. (24) 2018	Maxillary arch: 5%	Maxillary arch: 6%	12 persons ^*^	9 persons*	NS	Patients treated with aligners achieved satisfactory results, with no significant differences compared to patients treated with brackets.
	Mandibular arch: 11%	Mandibular arch: 11%	6 persons**	3 persons**		
Lin et al. (25) 2022	Total alignment: 3.5 (2.0; 5.5	3.0 (2.0; 5.75)	-	-	.993	There were no significant differences between groups in post-treatment changes in total OGS scores.
	Marginal ridges: 1.5 (0.0; 2.0)	1.0 (0.0; 2.0)			.474	
	Buccolingual inclination: 1.0 (0.0 2.25)	2.0 (0.0; 2.0)			.586	
	Occlusal contacts: 0.0 (0.0; 2.25)	1.0 (0.0; 2.0)			.373	
	Overjet: 2.0 (1.0; 5.25)	3.0 (1.0; 4.75)			.740	
	Interproximal contacts: 0.0 (0.0; 0.0)	0.0 (0.0; 0.0)			.209	
	Root angulation: 0.0 (0.0; 0.1)	0.50 (0.0; 2.0)			.050	
	Occlusal relationship: 1.5 (0.0; 4.0)	2.5 (0.25; 5.0)			.289	
	OGS: 12.5 (8.0;17.25)	14.5 (9.25; 21.75)			.367	

## Data Availability

The datasets used and/or analyzed during the current study are available from the corresponding author.

## References

[1] Littlewood SJ, Kandasamy S, Huang G (2017). Retention and relapse in clinical practice. Aust Dent J.

[2] Alassiry AM (2019). Orthodontic Retainers: A Contemporary Overview. J Contemp Dent Pract.

[3] Yu Y, Sun J, Lai W, Wu T, Koshy S, Shi Z (2013). Interventions for managing relapse of the lower front teeth after orthodontic treatment. Cochrane Database Syst Rev.

[4] Ben Mohimd H, Bahije L, Zaoui F, Halimi A, Benyahia H (2018). Is systematic mandibular retention mandatory? A systematic review. Int Orthod.

[5] Papageorgiou SN, Koletsi D, Iliadi A, Peltomaki T, Eliades T (2020). Treatment outcome with orthodontic aligners and fixed appliances: a systematic review with meta-analyses. Eur J Orthod.

[6] Kartal Y, Kaya B (2019). Fixed Orthodontic Retainers: A Review. Turk J Orthod.

[7] Littlewood SJ, Dalci O, Dolce G, Holliday LS, Naraghi S (2021). Orthodontic retention: what's on the horizon?. Br Dent J.

[8] Díaz P, Aguilar J (2017). Tratamiento de la recidiva en un paciente con extracciones previas de primeros premolares. Revista Mexicana de Ortodoncia.

[9] Amer I, Oztas E, Marsan G (2019). Orthodontic treatment with clear aligners and the scientific reality behind their marketing: A literature review. Turk J Orthod.

[10] Kou Z, Yi Q, Zhi X (2021). Guidelines for clear aligner orthodontic treatment. Society of Orthodontics. Chinese Stomatological Association.

[11] Weir T (2017). Clear aligners in orthodontic treatment. Aust Dent J.

[12] Upadhyay M, Abu S (2021). Biomechanic of clear aligners: hiddens truths and first principles. Journal of the world federation of orthodontics.

[13] Upadhyay M, Arqub SA (2022). Biomechanics of clear aligners: hidden truths & first principles. J World Fed Orthod.

[14] Kou Z, Yi Q, Zhi X (2019). Clear aligner therapy: risks and clinical strategies. Zhonghua Kou Qiang Yi Xue Za Zhi.

[15] Saleh M, Hajeer MY, Muessig D (2017). Acceptability comparison between Hawley retainers and vacuum-formed retainers in orthodontic adult patients: a single-centre, randomized controlled trial. Eur J Orthod.

[16] Doomen R, Aydin B, Kuitert R (2018). Mogelijkheden en beperkingen van orthodontische behandeling met clear aligners. Een verkenning [Possibilities and limitations of treatment with clear aligners. An orientation]. Ned Tijdschr Tandheelkd.

[17] Yassir YA, Nabbat SA, McIntyre GT, David R Bearn (2022). Eficacia clínica del tratamiento con alineadores transparentes en comparación con el tratamiento con aparatos fijos: una descripción general de las revisiones sistémicas. Clin Oral Invest.

[18] Casetta M, Guarnieri R, Altieri F (2020). The combined use of clear aligners and computed-guided piezocision: a case report with a 2-year follow-up. Int J Comput Dent.

[19] Ke Y, Zhu Y, Zhu M (2019). A comparison of treatment effectiveness between clear aligner and fixed appliance therapies. BMC Oral Health.

[20] Wells G, Shea B, O'Connell D, Peterson J, Welch V, Losos M (2024). The Newcastle-Ottawa Scale (NOS) for assessing the quality of nonrandomised studies in meta-analyses.

[21] Sterne JAC, Savović J, Page MJ, Elbers RG, Blencowe NS, Boutron I (2019). RoB 2: A revised tool for assessing risk of bias in randomised trials. BMJ.

[22] Graf I, Puppe C, Schwarze J, Hofer K, Christ H, Braumann B (2021). Evaluation of effectiveness and stability of aligner treatments using the Peer Assessment Rating Index. J Orofac Orthop.

[23] Kuncio D, Maganzini A, Shelton C, Freeman K (2007). Invisalign and traditional orthodontic treatment postretention outcomes compared using the American Board of Orthodontics objective grading system. Angle Orthod.

[24] Lanteri V, Farronato G, Lanteri C, Caravita R, Cossellu G (2018). The efficacy of orthodontic treatments for anterior crowding with Invisalign compared with fixed appliances using the Peer Assessment Rating Index. Quintessence Int.

[25] Lin E, Julien K, Kesterke M, Buschang PH (2022). Differences in finished case quality between Invisalign and traditional fixed appliances. Angle Orthod.

[26] Rossini G, Parrini S, Castroflorio T, Deregibus A, Debernardi CL (2016). Diagnostic accuracy and measurement sensitivity of digital models for orthodontic purposes: A systematic review. Am J Orthod Dentofacial Orthop.

